# Interspecific discovery and expression profiling of *Eucalyptus *micro RNAs by deep sequencing

**DOI:** 10.1186/1753-6561-5-S7-P173

**Published:** 2011-09-13

**Authors:** Marília Pappas, Alessandra Reis, Laurent Farinell, Giancarlo Pasquali, Georgios Pappas, Dario Grattapaglia

**Affiliations:** 1 Embrapa Genetic Resources and Biotechnology, Brazil; 2 Catholic University of Brasília, Brazil; 3 Fsateris SA, Brazil; 4 Federal University of Rio Grande do Sul, Brazil

## Background

Micro RNAs (miRNAs) are a class of small (~21 nucleotide) non-coding RNAs that recently gained much attention due to their perceived role as master regulators of gene expression in Eukaryotes, responsible for fine tuning gene expression regulation and, in plants, has been shown to be involved in a diverse range of biological processes such as plant development and architecture, flowering, cell differentiation and response to biotic and abiotic stresses [1]. The repertoire of expressed miRNAs differs among cell types, tissues, development, environmental condition, etc [2]. Notwithstanding, the exact function of thousands miRNAs sequences present in miRBase [http://www.mirbase.org/] is not elucidated. At this point, discovery and profiling of new and conserved miRNAs are critical in the attempt to understand their function and mechanism. Deep sequencing through next generation sequencing is the methodology of choice for this purpose as its ultra high throughput permits a comprehensive interrogation of the small RNA transcriptome, permitting *de novo* identification and relative quantification of different small RNA species [3].

Due to its economic importance the *Eucalyptus grandis* genome has been sequenced by JGI and the annotation of miRNAs is pivotal. In order to provide the first large scale experimental characterization of *Eucalyptus* miRNAs we performed an Illumina deep sequencing run that allowed us to discover and quantify the miRNA levels in two different tissues – xylem and leaves. Additionally, to get insights of the observed phenotypic differences in wood quality among *Eucalyptus* species, we characterized the xylem small RNA transcriptome of two different *E. globulus* individuals and integrated the results to catalog conserved and *Eucalyptus* specific miRNA gene families.

## Materials and methods

Four biological samples were used: xylem from two *E. globulus* genotypes, xylem and leaf from BRASUZ1 *E. grandis*, the one currently being sequenced by JGI. Total RNA extraction was performed with CTAB protocol to a total amount of 10 mg per sample [4]. Fraction of small RNAs were barcoded to be sequenced in a single flow cell in Illumina GA II Sequencing System by Fasteris [http://www.fasteris.com]. A computational pipeline was specifically developed to process the deep sequencing data. The pre-processing step cleans the sequences by quality screening, adapter sequence removal, contaminant checking. Cleaned reads were sorted according to size, quantified (tag counting) and used to create an additional set of non-redundant sequences (using uclust). Bowtie was used to map sequences against the 8X *E. grandis* – BRASUZ1 genotype – genome sequence draft. Mapped positions in the genome were extended by 150 bases to be used as input to predict secondary structure (miRDeep) to test for stem loop structure of miRNA precursors. Northern blot hybridization is being used for experimental validation of some conserved and potentially new *Eucalyptus* miRNAs sequences.

## Results and conclusions

Total number of reads was 6,104,498 ranging from 1,115,404 to 1,766,355 per sample – 36 nt average size. After pre-processing, total number of sequences was reduced to 1,980,958. As expected, read size distribution has two main peaks at 21 and 24 nt. Comparative analysis of size distribution interestingly shows higher abundance of the 24 nt fraction for all samples, being up to 3,75 times higher than 21 nt. The 24 nt small RNAs are predominantly small interfering RNAs (siRNAs) which are involved in RNA-directed DNA methylation resulting in gene and transposon silencing. Putting it all together, reads from four samples resulted in 169,642 unique sequences mapped against the genome. From that, 70,55% had at least one alignment to the genome reported, 23,54% failed to align and 5,91% mapped to multiple loci, indicative of repeat regions.

Mapping 20-22 nt reads against the reference genome revealed that BRASUZ1 had more reads mapping to its own genome than the other samples, totaling 95% for leaves sample and 91% for the xylem. *E. globulus* samples showed a reduced percentage of mapped reads, around 85%, corroborating the existence of interspecific variability. Besides that, the relative abundance among the three xylem samples reinforces the variability also in the expressed repertoire of small RNAs.

Annotation of plant miRNAs was done meeting a set of strict criteria, particularly a proper secondary structure of the precursor [5]. The positions in the genome with a mapped reads were extended to 300 bases and fed to the program miRDeep [6] to test for the compatibility of precursor hairpin. At least 38 sequences mapped were positive under these premises.

Conserved *Eucalyptus* miRNAswere identified by similarity searches against the miRBase. A total of 206 distinct *Eucalyptus* sequences showed significant similarity (at most one mismatch) to an orthologous sequence, confirming the presence of 36 different mir genes families, including many of its isoforms.

Quantitative differences in miRNA abundance were probed by pairwise comparison of tag counts contrasting intraspecific, interspecific and tissue-specific analysis. Results revealed that the most similar small RNA repertoire are between intraspecific samples and, the least, between tissue specific. Tissue specific differential expression analysis shows that around 36% of the conserved miRNAs sequences observed in each tissue was mutually exclusive and the ones present in both samples vary up to two-fold (p-value=0.05).

Experimental validation is being carried out by northern blot hybridization and preliminary results validated new and conserved miRNAs, such as mir 156 and mir 172.
